# Transport processes and coalescence of two entrapping bubbles during upward solidification

**DOI:** 10.1016/j.heliyon.2025.e42669

**Published:** 2025-02-12

**Authors:** I.C. Hsieh, P.T. Tseng, W.T. Chen, P.S. Wei, K.C. Liao

**Affiliations:** Department of Mechanical and Electro-Mechanical Engineering, National Sun Yat-Sen University, Kaohsiung, 80424, Taiwan, ROC

**Keywords:** Solute segregation, Pore coalescence, Bubble merging, Pore shapes, Solidification, Lotus-type pores

## Abstract

The transport processes and coalescence of a pair of bubbles during upward unidirectional solidification of water containing carbon dioxide are numerically investigated. In addition to affecting material microstructure, functional porous materials have applications in biology, tissue engineering, food preservation, and addressing global warming through porous sea ice. In this study, transport equations within the bubble, liquid, and solid phases are solved using the commercial COMSOL software (version 5.2). The results indicate that coalescence is promoted by fluid flow after bubbles make contact. Coalescence is likely when the concentration at the midpoint between two bubbles, each with identical concentrations, matches the concentration within the bubbles over a short distance. It is observed that bubble coalescence is enhanced by increases in horizontal incoming velocity, surface tension, and liquid solute diffusivity, as well as by decreases in Henry's law constant, ambient pressure, partition coefficient, and solid thermal conductivity. The solute concentration around pores also increases with decreasing Henry's law constant and liquid solute diffusivity, and increasing horizontal velocity, ambient pressure, solid thermal conductivity, and surface tension. The predicted contact angle during solidification aligns well with Abel's equation. The development of multiple pores and solute segregation inevitably occurs, influencing the material's microstructure. This can be managed by analyzing all the transport and metallurgical properties and adjusting the related working parameters accordingly.

## Highlights

• Transport processes, coalescence and entrapment of two bubbles during upward unidirectional solidification are numerically investigated.
• Coalescence is promoted by fluid flow after bubbles make contact.
• Bubble coalescence affected by transport parameters are presented.
• Distributions of solute concentration surrounding the pores are presented.

## 1 Introduction

Porous structural and functional materials are prevalent in welding [1,2], casting [3], additive manufacturing [4], phase change materials [5], and atmospheric sciences [6]. Pore shapes smaller than the solidification front morphology occur in water [7,8], pure metals [9], and eutectic alloys [10]. The performance of these structures hinges on understanding the growth, interruption, and coalescence of the pores during solidification processes [[10], [11], [12]]. The mechanisms of bubble growth and coalescence have been numerically explained as being attributed to fluid flow, pressure differences, and the thickness of the film between bubbles encountered during cavitation processes [13,14]. This forms the aim of this work, which addresses the transport processes involved in the development, coalescence, and entrapment of two bubbles during upward solidification.

Shapovalov [15] schematically depicted the pore nucleation, pore interruption, pore coalescence, diameter change, and orientation change of pores in directionally solidified porous metals. In view of difference in pressures between two pores, Ide et al. [16] and Song et al. [17] mentioned that coarse pores were evolved by coalescing small pores. Drenchev et al. [18] proposed that pore coalescence was aimed at obtaining a structure of lower energy. Murakami and Nakajima [7] also observed the coalescence of columnar pores during unidirectional solidification of water containing carbon dioxide. Liu et al. [11] used X-ray tomography to observe the pore growth process in the Gasar metals, which can be divided into initial, steady-state growth, and interruption stages. It was qualitatively interpreted by comparing the rates of change in solute gas pressure and the apex radius of the pore, resulting from solute transport into the pore. The shape evolution of pore coalescence process can also be geometrically divided into five stages: (a)starting state; (b) externally tangent state; (c) common tangent state; (d) internally tangent state; (e) ending state. For the Gasar metals with high porosity, the pore coalescence becomes prominent.

Liu et al. [10] observed the nucleation, interruption, coalescence, diameter changes of pores in actual pore structures. Pore nucleation and interruption increased pore number density while reducing interpore spacing, whereas pore coalescence increased average pore diameter and decreased pore number density. Similar to eutectic growth, rod elimination increases rod spacing, while splitting decreases it. The coupled growth of gas and solid phases was proposed to be governed by solute diffusion and interface response due to capillary and kinetic effects. When interpore spacing is small, the interface energy rises, leading to pore coalescence or interruption, which reduces interface energy. When interpore spacing is large, hydrogen was considered to accumulate away from pores, promoting nucleation in solute-enriched zones. Liu et al. [19] also experimentally found that a higher superheat can cause the pores coalescence during Gasar solidification. Many big pores therefore arise at the bottom and the top of the ingot and a non-uniform porous structure forms.

This study, building upon a previous model [20], systematically and quantitatively predicts solute concentration, velocity fields, and the development, coalescence, and entrapment of a pair of bubbles by an upward-moving planar solidification front. The effects of mechanical, thermal, and metallurgical properties on formation and microstructure, including the final pore shapes and solute segregation around pores within the solid, are presented.

## 2 System model and governing equations

**Fig. 1:**
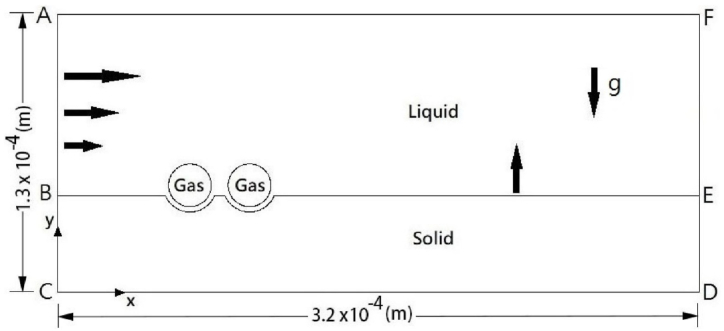
Physical model.

This model addresses the entrapment of a pair of bubbles on a solidification front moving upward, with gravity acting in the downward direction, as illustrated in Fig. 1. The width and height of the physical domain selected are $3.2\times 10^{-4}$ and $1.3\times 10^{-4}$ m, respectively. Cartesian coordinates are selected with the origin at the intersection between the left boundary BC and bottom boundary CD. Each bubble has an initial radius of $10^{-5}$ m at a solidification front location of y = $4.5\times 10^{-5}$ m. Two bubbles are located with x-y coordinates of $6.5\times 10^{-5}$ and $5\times 10^{-5}$ m, and $1.05\times 10^{-4}$ and $5\times 10^{-5}$ m, respectively. Incoming velocity is imposed on the left boundary AB. The upward solidification is proceeded due to a cold temperature imposed at the bottom boundary CD. The Henry's law is satisfied at liquid-gas interface AF and bubble cap.

Without loss of generality, it is assumed that a pair of bubbles emerges from an approximately flat solidification front at an initial time. The interpore spacing is specified, although it can be scaled and is a consequence of morphological instability [21]. The primary limitation of this assumption is the use of a two-dimensional model, where variations in the z-direction are considered negligible. Instead of a complex three-dimensional study, the bubbles are assumed to be cylindrical in shape. This simplified model can be regarded as a first approximation, allowing for a more rigorous and systematic understanding of the complex physical mechanism to be achieved. Additionally, the pores contain only solute gas, with any vaporized gas from the solvent being negligible. The governing equations of mass, momentum applicable to distinct phases [20] are, respectively,

(1) $$\frac{\partial \rho }{\partial t}+\nabla \cdot \rho v=0$$

(2) $$\rho \left(\frac{\partial v}{\partial t}+v\cdot \nabla v\right)=-\nabla p+\nabla \cdot \left\{\mu \left[\nabla v+{\left(\nabla v\right)}^{T}\right]-\frac{2}{3}\mu \left(\nabla \cdot v\right)I\right\}+\rho g+\left\{-\sigma \kappa n+\frac{\partial \sigma }{\partial T}\left[\nabla T-\left(n\cdot \nabla T\right)n\right]+\frac{\partial \sigma }{\partial C}\left[\nabla C-\left(n\cdot \nabla C\right)n\right]\right\}\cdot I\delta \left(n-n_{s}\right)$$

where the terms on the last bracket capillary pressure, and Marangoni forces due to temperature (namely, theromcapillary force) and concentration gradients of Eq. (2), respectively. Energy equation yields

(3) $$\frac{\partial \rho c_{p}T}{\partial t}+\nabla \cdot \rho c_{p}vT=\nabla \cdot \left(k\nabla T\right)-\left(\frac{\partial \rho L_{total}}{\partial t}+\nabla \cdot \rho vL_{total}\right)$$

where the last terms represent the temporal change and the convective transport of latent heat evolution, respectively. The latent heat $L_{total}$ includes enthalpy due to difference in averaged specific heats over the temperature ranges considered between liquid and solid and the maximum latent heat at the melting temperature [22]. Concentration equation is

(4) $$\frac{\partial C}{\partial t}+\nabla \cdot vC=\nabla \cdot \left(D\nabla C\right)-\left(1-k_{p}\right)Cf_{l}\frac{\partial f_{l}}{\partial t}Vf_{mat}-\left(\frac{p_{g}}{R_{u}T}-\frac{p_{g}}{K}\right)\left(v-V_{in}\right)\cdot n\delta \left(n-n_{lg}\right)Vf_{mat}+C_{const}Vf_{g}\left(\frac{p_{g}}{R_{u}T}-C_{g}\right)$$

where the solute convection on the left-hand side is balanced by the solute diffusion, segregation at the solidification front and bubble cap, and the equation of state in the pore on the right-hand side, respectively. Choosing an arbitrarily high value of $C_{const}$ the last term reduces to the equation of state. Local properties applicable for distinct phases in Eqs. (1), (2), (3), (4) are

(5) $$\left(\begin{array}{l} \rho \\ k\\ \mu \\ c_{p}\\ D \end{array}\right)=Vf_{g}\left(\begin{array}{l} \rho_{g}\\ k_{g}\\ \mu_{g}\\ {c_{p}}_{g}\\ D_{g} \end{array}\right)+Vf_{mat}\left[\left(\begin{array}{l} \rho_{s}\\ k_{s}\\ \mu_{s}\\ c_{ps}\\ D_{s} \end{array}\right)+f_{l}\left(\begin{array}{l} \rho_{l}-\rho_{s}\\ k_{l}-k_{s}\\ \mu_{l}-\mu_{s}\\ c_{pl}-c_{ps}\\ D_{l}-D_{s} \end{array}\right)\right]$$

where liquid-gas interface can be described from the phase field equation [23].

(6) $$\frac{\partial \phi }{\partial t}+\left[v+\left(V_{in}-v\right)\right]\cdot \nabla \phi =\nabla \cdot \left(\frac{\omega \lambda }{\varepsilon^{2}}\nabla \psi \right)$$

(7) $$-\nabla \cdot \left(\varepsilon^{2}\nabla \phi \right)+\left(\phi^{2}-1\right)\phi \equiv \psi$$

The difference in velocities between the interface and liquid in Eq. (6) is given by [24].

(8) $$V_{in}-\nu =\frac{-D\left(n\cdot \nabla C\right)}{\left(\frac{p_{g}}{R_{u}T}-\frac{p_{g}}{K}\right)}$$

which indicates that solute transfer across a moving interface is due to relative velocities between the liquid and interface, and solute diffusion in liquid. Equations (1), (2), (3), (4), (6), and (7), together with Equations (5), (8), were solved using COMSOL Multiphysics version 5.2, with the variable χ≤ 0.01 and interface thickness ε = $7\times 10^{-7}$ m. The relative error, using a total of $10^{6}$ meshes (as will be shown later), was less than 0.01. The initial conditions for concentration were 50, 35, and 0.01 $\text{mol}/m^{3}$ in the gas, liquid, and solid phases, respectively. The gas pressure in the pore was 1.06 atm, and the temperatures were 273.5 K for the gas, 274 K for the liquid, and 265–273 K for the solid. The boundary conditions are provided in Table 1.

**Table 1:** Boundary conditions.

Boundary	$C\;\left(mol/m^{3}\right)$	p(atm)	T(K)	(u,v)(m/s)
AB	$\vec{n}\cdot D_{l}\nabla C_{l}=0$	1	$\frac{\partial T}{\partial x}=0$	(0.04,0) or *(0.01,0)*
BC	$10^{-3}$, $10^{-2}$	1	$\frac{\partial T}{\partial x}=0$	−
CD	$10^{-3}$, $10^{-2}$	1	265, 271	−
DE	$10^{-3}$, $10^{-2}$	1	$\frac{\partial T}{\partial x}=0$	−
EF	$\vec{n}\cdot D_{l}\nabla C_{l}=0$	1	$\frac{\partial T}{\partial x}=0$	−
AF	$\frac{1}{K}\left(p_{a}+\rho gh+\frac{2\gamma }{R_{0}}\right)$	1	275,276	−

## 3 Results and discussion

**Fig. 2:**
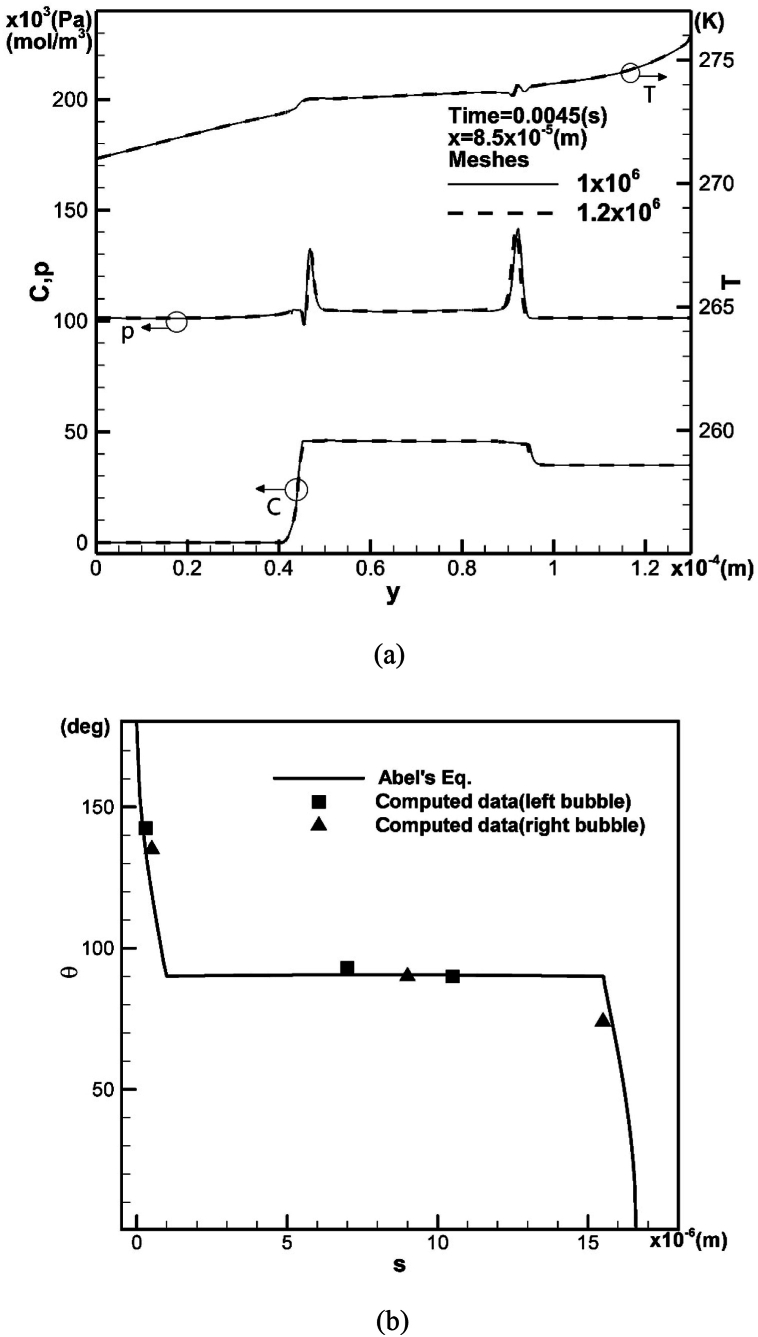
(a) Effect of meshes on predicted vertical concentration, pressure and temperature distributions through horizontal location x = 8.5 $\times 10^{-5}$ m between two bubbles, and (b) contact angle versus location of solidification front by comparison with that obtained from Abel's equation.

This study proposes a general and systematic model to investigate transport variables during the merging process of a pair of bubbles entrapped in an upward solidification process. The development of multiple pores and solute segregation inevitably occurs, influencing the material's microstructure. This can be controlled by examining transport and metallurgical properties and adjusting working parameters. Fig. 2(a) shows the effect of mesh resolution on the predicted vertical concentration, pressure, and temperature distributions along the horizontal location x = $8.5\times 10^{-5}$ m between two bubbles at a time of 0.0045 s. The left y-axis represents pressure and concentration, while the right y-axis represents temperature. A coarser mesh grid of $10^{6}$ is used, as it is sufficiently accurate to achieve converged results. Fig. 2(b) shows that the predicted contact angle as a function of the location of the solidification front aligns well with the results obtained from Abel's equation of the first kind [25], which were validated by experimental results [8] and theoretical predictions from fluid flow [26,27]. However, the maximum deviation of 10 % can be observed in the case of [28] between Abel's equation and the experimental data [8] near the early stage of solidification. Since the two bubbles usually grow at the same rate and over the same time period, the data for comparison is considered sufficient.

**Fig. 3:**
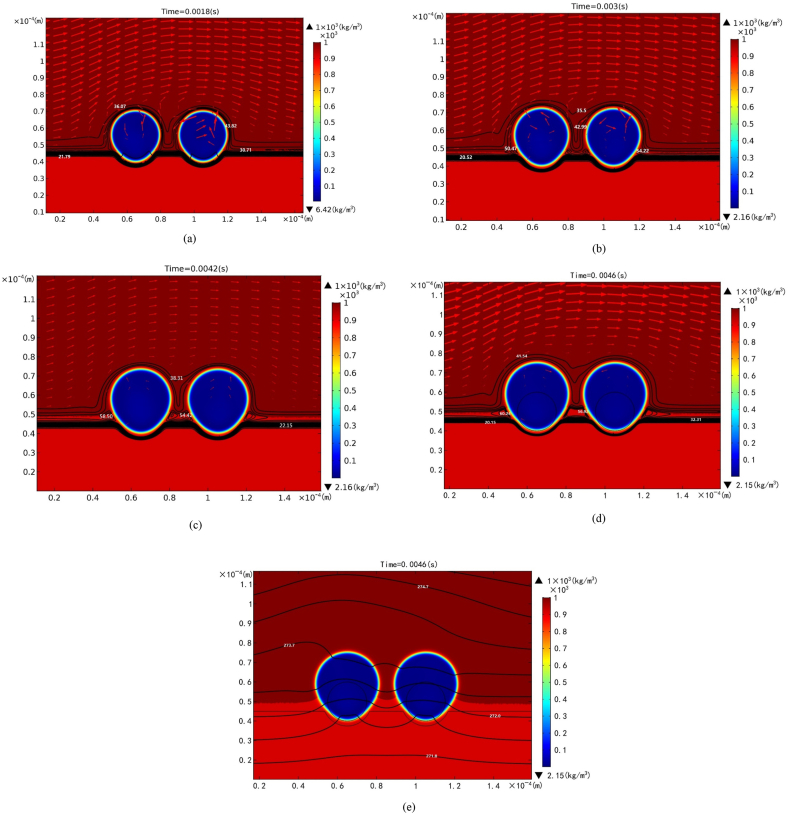
Pore shapes together with velocity field and iso-concentration (in unit of $\text{mol}/m^{3}$) at (a) 0.0018 s, (b) 0.003 s, (c) 0.0042s, and (d) 0.0046 s, and isotherm at time of (e) 0.0046 s subject to ambient pressure $p_{a}$ = 1atm and horizontal liquid velocity u = 0.04 m/s imposed on left boundary, and Henry's law constant K = 2900 $\text{Pa}-m^{3}/\text{mol}$, surface tension σ = 0.076 N/m, inter-pore spacing *w* = $4\times 10^{-5}$ m, partition coefficient $k_{p}$ = 0.1, solid thermal conductivity $k_{s}$ = 2.3 W/m-K, and liquid solute diffusivity $D_{l}=10^{-9}\;m^{2}/s$.

Fig. 3(a) shows the pore shape together with iso-concentration and velocity fields subject to horizontal velocity u = 0.04 m/s at left boundary at time of 0.0018 s. Other working parameters are ambient pressure $p_{a}$ = 1atm imposed on left boundary, and Henry's law constant K = 2900 $\text{Pa}-m^{3}/\text{mol}$, surface tension σ = 0.076 N/m, partition coefficient $k_{p}$ = 0.1, inter-pore spacing *w* = $4\times 10^{-5}$ m, solid thermal conductivity $k_{s}$ = 2.3 W/m-K, and liquid solute diffusivity $D_{l}=10^{-9}\;m^{2}/s$.The white line stands for the pore boundary, whereas red, orange and blue represent fluid, solid and gas phases, respectively. The iso-concentration lines follow the shapes of the solidification front and the two bubbles. Dense variations in the iso-concentration lines indicate significant solute segregation around the bubble cap, pore boundary, and solidification front. The concentration in the bubbles, however, remains relatively constant, as can also be seen later. In view of the imposed horizontal velocity at the left boundary, the fluid flow is primarily around the caps of the two bubbles from the left to right sides. Velocity increases with distance from the bubble cap and the solidification front, with the thickness of the velocity boundary layer being approximately equal to the bubble radius, consistent with scale analysis of $\delta ∼\sqrt{\nu R/u_{\infty }}∼\sqrt{10^{-7}\times 10^{-5}/10^{-2}}\approx 10^{-5}$ m [26]. It is noted that a small upward velocity exists in the gap between the two bubbles. The velocity field is similar as time progresses to 0.003 s, as shown in Fig. 3(b). However, a high concentration of around 54 $mol/m^{3}$ in a region with a triangular shape, covering the solid and liquid near the triple-phase line, develops between the leading and trailing edges of each bubble and the solidification front [26,27]. This is attributed to segregation from the solidification front and solute transport from the bubble. These concentration cells grow as time increases to 0.0042 s, as depicted in Fig. 3(c). In addition to the continuous growth of concentration cells, the two concentration cells within the gap between the leading and rear bubbles merge into a single region at a time of 0.0046 s, as shown in Fig. 3(d). Fig. 3(e) also shows that the isotherms are primarily parallel to the solidification front. The isotherms become perpendicular to the surface of the bubble due to the significant difference in thermal conductivities between the gas and the liquid or solid. As a result, the shape of the solidification front at an isothermal of 273 K near the bubble cap deforms upward and aligns perpendicular to the bubble cap. Consequently, the solidification rate can be affected by the shape of the bubble cap [26,27].

**Fig. 4:**
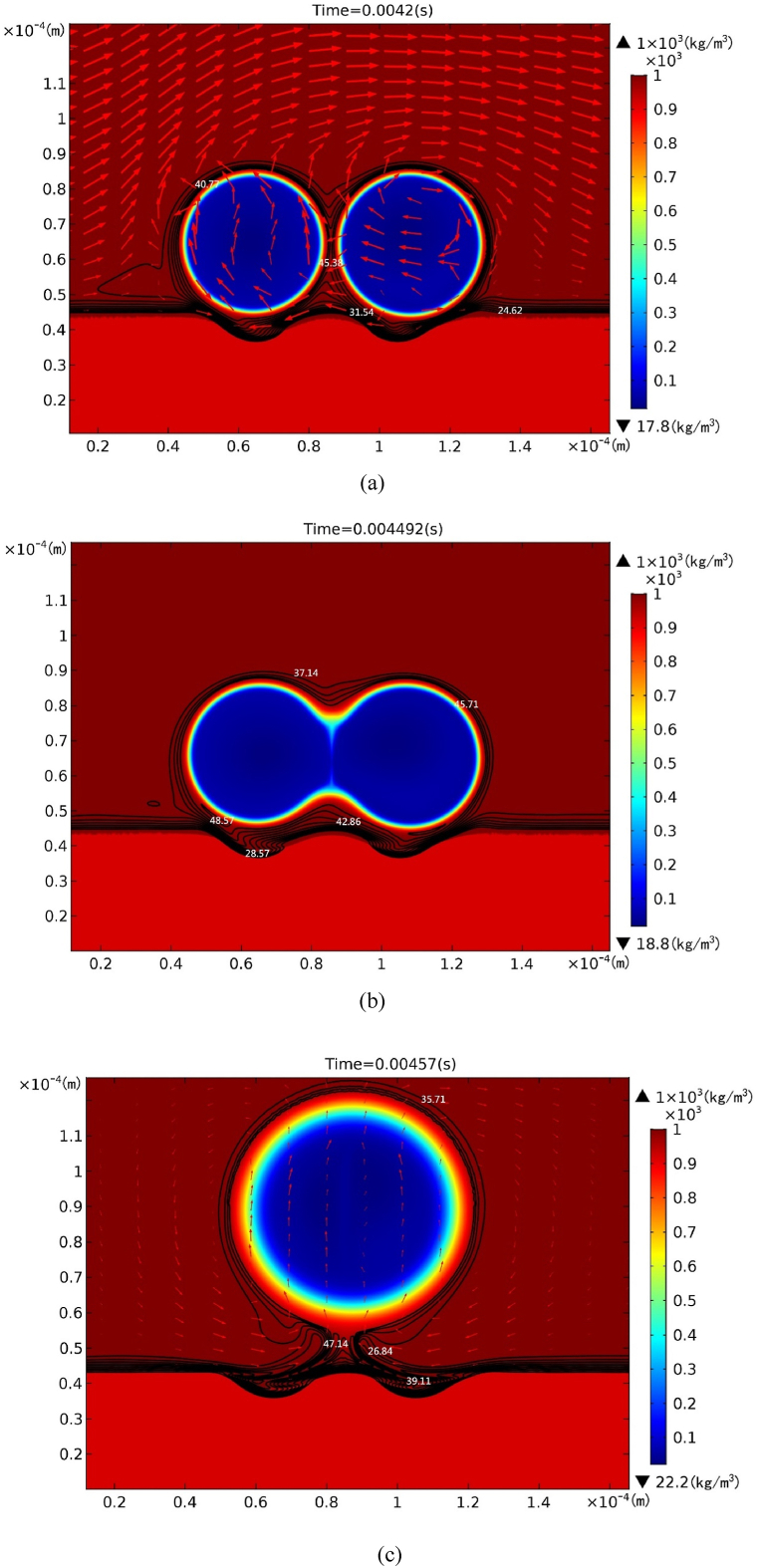
Coalescing two bubbles together with velocity field and iso-concentration (in unit of $\text{mol}/m^{3}$) at (a) 0.0042 s, (b) 0.004492 s, and (c) 0.00457 s for solid thermal conductivity $k_{s}$ = 0.23 W/m-K.

Fig. 4(a) shows pore shapes together with velocity and iso-concentration at time of 0.0042 s as solid thermal conductivity decreases to 0.23 W/m-K. Due to the slow solidification rate caused by low solid thermal conductivity, the bubbles can be considered to grow without influence from the solidification front. The difference in hydrostatic pressure on the bubble caps causes the two bubbles to float upward, creating a thin liquid layer between the bubbles and the solidification front. In this region, the direction of the flow and the iso-concentration lines follow the shape of the bubble caps. The two growing bubbles are comparatively rapidly merged, as shown in Fig. 4(b). Coalescence of two bubbles gives rise to a big bubble, as shown in Fig. 4(c). The rising bubble creates a complex interaction region between opposing directions of velocity and iso-concentration in the liquid near the bottom.

**Fig. 5:**
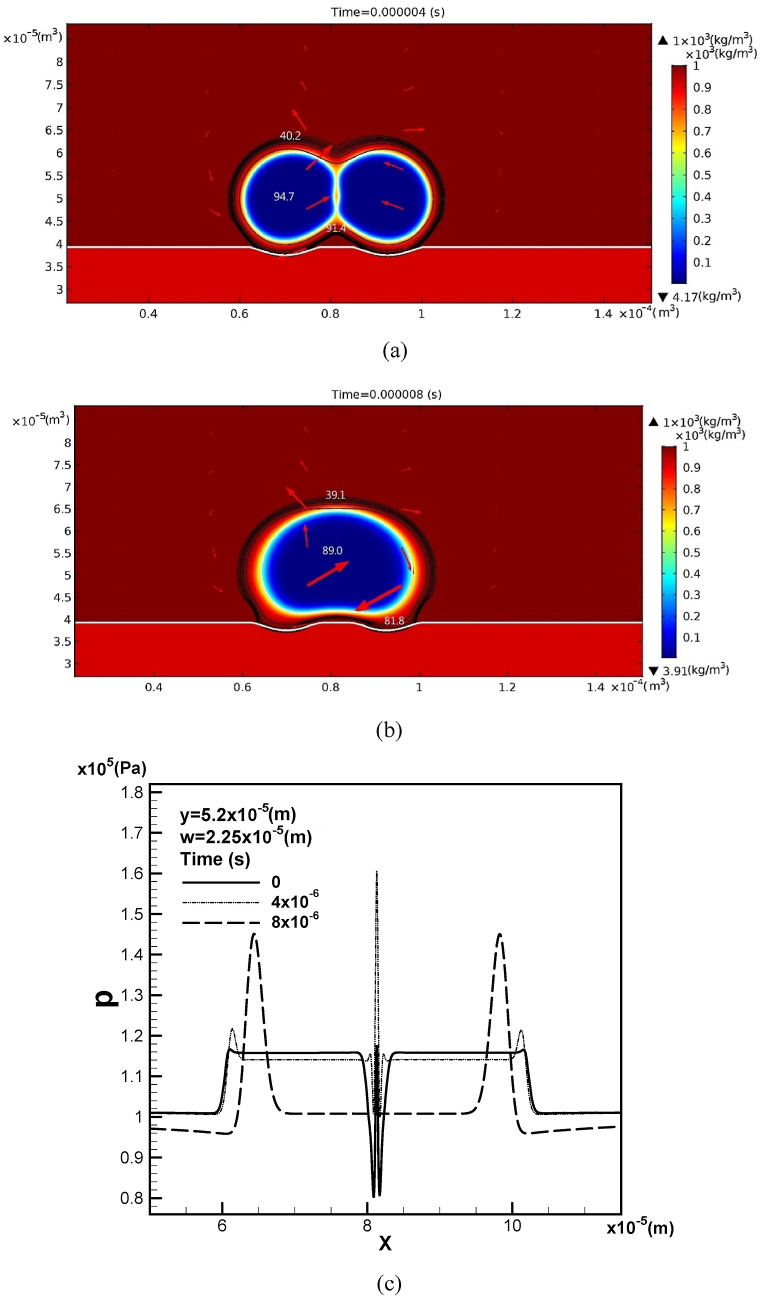
Coalescing two bubbles together with iso-concentration (in unit of $\text{mol}/m^{3}$) and velocity fields at (a) $4\times 10^{-6}$ s, (b) $8\times 10^{-6}$ s, and the corresponding (c) pressure profile along a horizontal line at y = $5.2\times 10^{-5}$ m with an inter-pore spacing *w* = $2.25\times 10^{-5}$ m.

Two bubbles are prone to coalescence, as indicated by the decreasing inter-pore spacing to $2.25\times 10^{-5}$ m over time, as shown in Fig. 5(a) and (b). Fig. 5(c) shows that the solute gas pressure in the leading and trailing bubbles is nearly identical, with peak values at the bubble boundary at different times. The peak pressures can reach $1.22\times 10^{5}$ Pa and $1.45\times 10^{5}$ Pa at a time of $4\times 10^{-6}$ and $8\times 10^{-6}$ s, respectively. The liquid pressure within the gap between the two bubbles is lower than the pressure inside the bubbles at different times. As time progresses, the liquid pressure decreases while the peak values increase. The pressure discontinuity between the two bubbles suddenly disappears upon coalescence at time of $8\times 10^{-6}$ s.

**Fig. 6:**
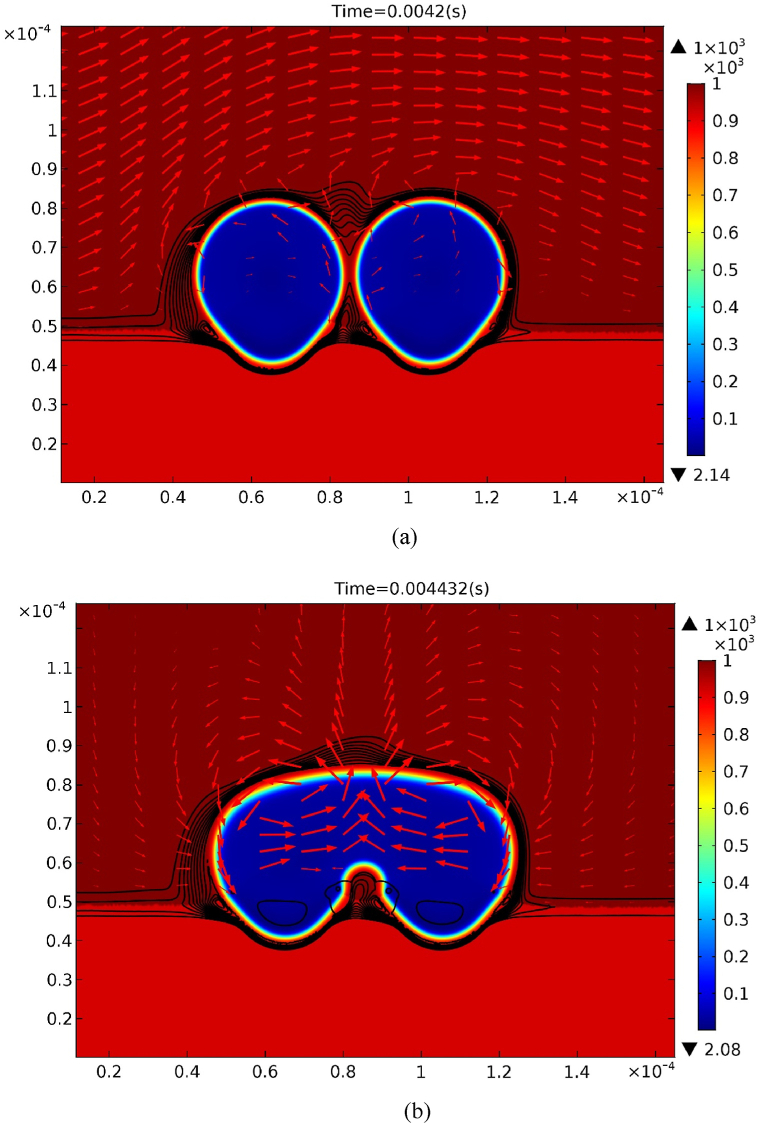
Coalescing two bubbles together with iso-concentration and velocity fields at (a) 0.0042 s, and (b) 0.004432 s subject to mole fraction γ = 0.5 at the free surface.

Two bubbles can easily be merged as the mole fraction at the top free surface decreases to 0.5, as shown in Fig. 6(a) and (b). It reveals that coalescence can be divided into two steps, namely, point contact and rapid merging. As two bubbles contact, the gap is separated into two regions. In contrast to small downward velocity in the lower region, velocity in the upper region jumps and directs toward the up direction. This is attributed to the solute gas pressure in bubbles maintaining a high value instantaneously after coalescence. The induced upward flow accompanied with bubble growth enhances a further rapid merging. The timescale or the mechanism of point contact and rapid merging can thus be related to the mass conservation of the local flow induced by the capillary pressure difference between the gap of the coalescing bubbles [13,14]. Further study on this topic is challenging. Flow circulates in the pore and surrounding liquid counterclockwise and clockwise through the leading and rear bubbles, respectively.

**Fig. 7:**
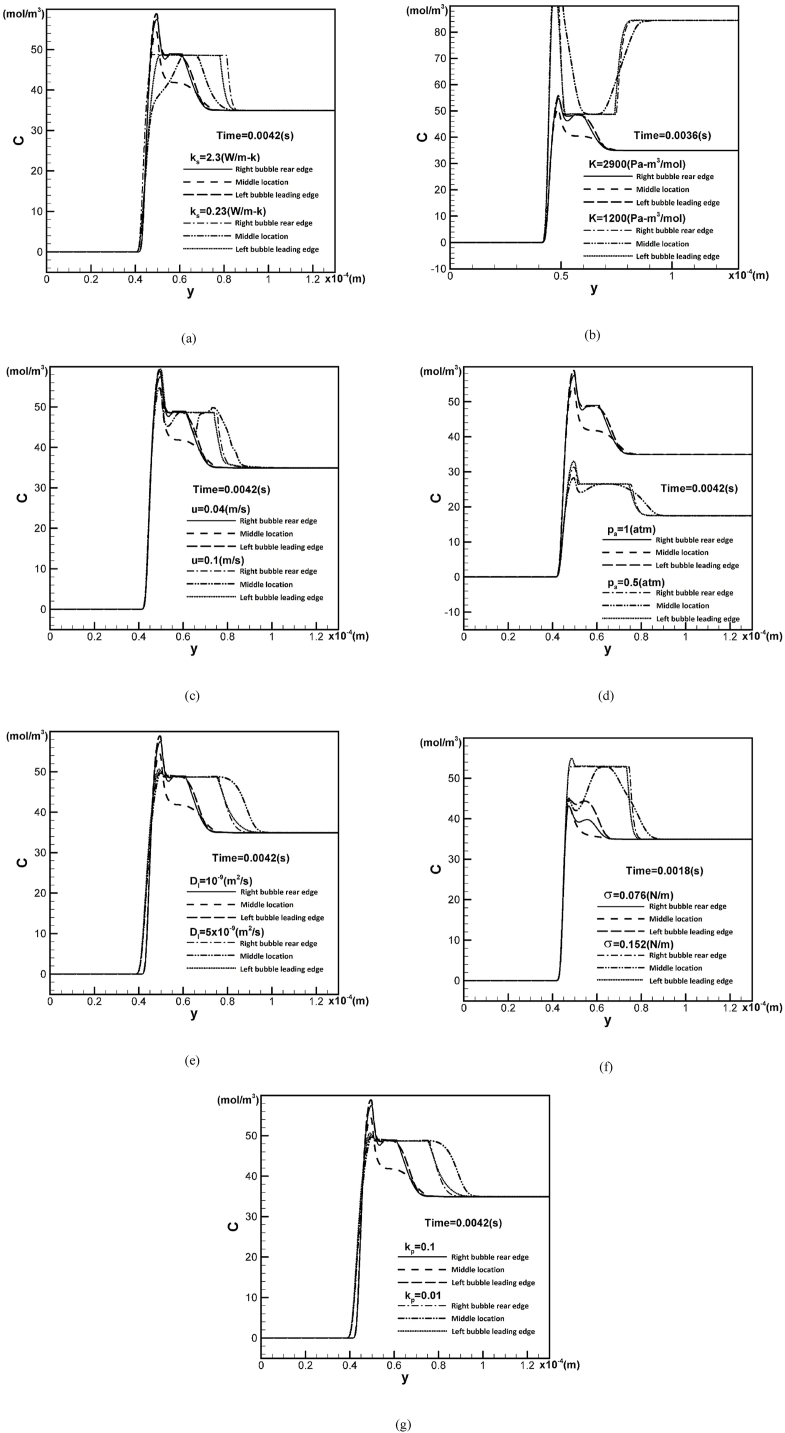
Concentration profiles through the center of the leading pore, at a location between two pores, and at the center of the rear pore for different values of (a) solid thermal conductivity, (b) Henry's law constant, (c) incoming velocity at the left boundary, (d) ambient pressure, (e) liquid solute diffusivity, (f) surface tension, and (g) partition coefficient.

Fig. 7(a) shows vertical concentration profiles through the three triple-phase lines: at the leading edge of the leading bubble, the middle location of the two bubbles, and the rear edge of the rear bubble, located at x = $4.8\times 10^{-5}$, $8.5\times 10^{-5}$, and $1.22\times 10^{-4}$ m, respectively, for different solid thermal conductivities at a time t = 0.0042 s. It can be seen that the concentration through the triple-phase line region in the leading edge of the leading pore is higher for a higher solid thermal conductivity of 2.3 W/m-K. A constant plateau of concentration around 48 $\text{mol}/m^{3}$ indicates the concentration in each bubble or merging bubbles. A higher solid thermal conductivity results in an increased cooling rate, which enhances the solidification rate, as described by the Stefan boundary condition at the solidification front [1]. Solute segregation or rejection by the solidification front therefore increases with the solidification rate. The concentration rapidly increases and follows by a decrease when passing through the triple-phase line region, the bubble, and the liquid. However, the concentration at the middle location at $8.5\times 10^{-5}$ m does not share with the concentration of 48 $\text{mol}/m^{3}$ of the bubble over a short distance, indicating that the two bubbles are not merged. In contrast, for a lower solid thermal conductivity of 0.23 W/m-K, the concentration increases and then maintain relatively constant, and decreases when passing through the triple-phase line region, the bubble, and the liquid regions. The concentration profile through the middle location between the two bubbles reaches and remains a short distance on the plateau, implying that the two bubbles have coalesced.

Regardless of the cutoff curve for better visualization, Fig. 7(b) shows that the concentration along the triple-phase line in the solid increases as Henry's law constant decreases to 1200 $\text{Pa}-m^{3}/\text{mol}$ at a time of 0.0036 s. Selecting different times (see Fig. 7(a)) is based on the fact that the merging time can vary depending on the working parameters. Due to the intersection of concentration at the midpoint between the two bubbles and within the pore over a short distance, the bubbles merge at a lower Henry's law constant of 1200 $\text{Pa}-m^{3}/\text{mol}$ at t = 0.0036 s.

As the incoming horizontal velocity increases to 0.1 m/s, the bubbles more readily coalesce, as depicted in Fig. 7(c). Solute concentration through the triple-phase line region is higher for greater incoming velocity of 0.1 m/s. A decrease in ambient pressure to 0.5 atm, increases in liquid solute diffusivity to $5\times 10^{-9}\;m^{2}/s$ and surface tension to 0.152 N/m, and a decrease in partition coefficient to 0.01 also result in easier coalescence, as shown in Fig. 7(d)–(g). Solute concentrations through the triple-phase line region in the solid are higher for greater ambient pressure, lower liquid solute diffusivity, and higher surface tension and partition coefficient.

**Fig. 8:**
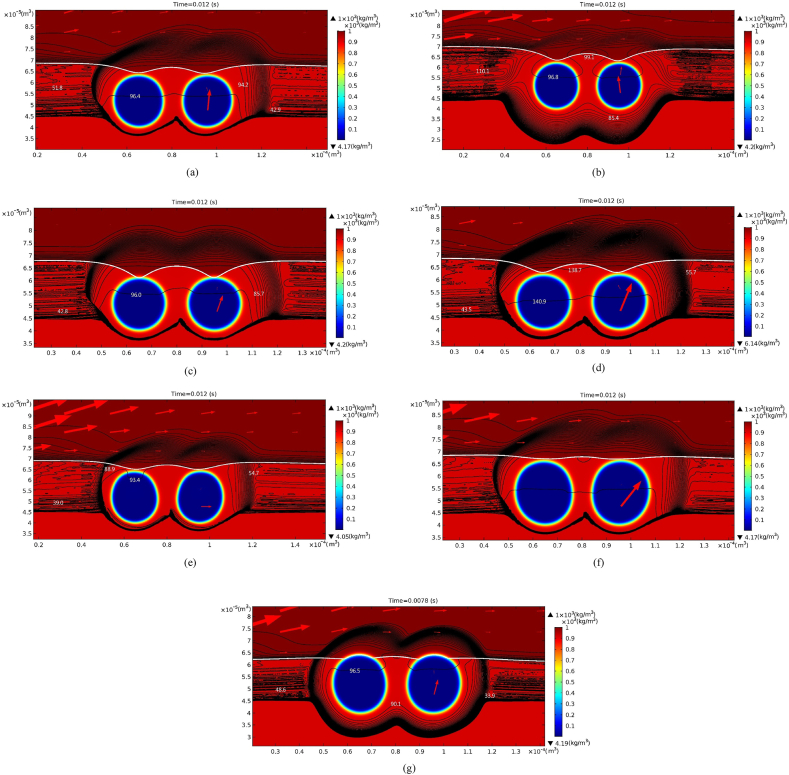
Iso-concentration (in unit of $\text{mol}/m^{3}$) around entrapped pores for (a) a typical case, (b) Henry's law constant K = 1450 $\text{Pa}-m^{3}/\text{mol}$, (c) incoming velocity u = $10^{-3}$ m/s, (d) ambient pressure $p_{a}$ = 2 atm, (e) surface tension σ = 0.038 N/m, and (f) partition coefficient $k_{p}=0.01$ at 0.012 s, and (g) liquid solute diffusivity $D_{l}=10^{-10}\;m^{2}/s$ at $7.8\times 10^{-3}$ s.

Concentration around entrapped pores is shown in Fig. 8(a). The parameters were chosen as K = 2900 $\text{Pa}-m^{3}/\text{mol}$, σ = 0.076 N/m, *u* = 0.01 m/s, $D_{l}$ = $10^{-9}$ $m^{2}/s$, $p_{a}$ = 1 atm, *w* = 3 $\times 10^{-5}$ m, and $k_{p}$ = 0.1. Referring to Fig. 7(a)–(g), the highest concentration due to segregation occurs around the bubbles and the solidification front at the initial time. The concentration gradient in the liquid ahead of the leading pore is higher than that at the rear edge of the trailing pore, due to the only asymmetric conditions created by the impact velocity from upstream location. Comparing to previous Fig. 8(a) and (b) shows that the concentration fields become symmetric with a smaller Henry's law constant K = 1450 $\text{Pa}-m^{3}/\text{mol}$ , as this reduces solute transport across the pores (see Eq. (8)). Similar asymmetric concentration fields in comparison with previous Fig. 8(a) are observed in Fig. 8(c)–(f) as incoming velocity decreases to 0.001 m/s, ambient pressure increases to 2 atm, surface tension decreases to 0.038 N/m, and partition coefficient decreases to 0.01, respectively. For the same reason related to a low Henry's law constant (see Fig. 8(b)), Eq. (8) also indicates that a smaller liquid solute diffusivity results in lower solute transfer across the bubbles and symmetric concentration fields in the leading and trailing regions of the bubbles, as shown in Fig. 8(g).

## 4 Conclusions

In this study, numerical predictions indicate that the flow circulates clockwise before merging, while it becomes counterclockwise around the leading bubble and clockwise around the rear bubble within the surrounding liquid on merging. The concentration gradient in the liquid ahead of the leading bubble is typically higher than that at the rear edge of the trailing bubble due to the impact velocity from upstream locations. However, for smaller Henry's law constants and lower liquid solute diffusivity, the concentration fields become more symmetric, as solute transport across the pores is reduced. The solute concentration in the solid and liquid surrounding the pore increases with decreasing Henry's law constant and liquid solute diffusivity, as well as with increasing incoming horizontal velocity, ambient pressure, solid thermal conductivity, surface tension, and partition coefficient. A pair of bubbles is more prone to coalescence with decreasing Henry's law constant, ambient pressure, solid thermal conductivity, and partition coefficient, and with increasing horizontal incoming velocity, surface tension, and liquid solute diffusivity. Once the coalesced bubbles rise, the upward motion creates a complex interaction region with opposing velocities and iso-concentration lines near the bottom, particularly as solid thermal conductivity or solidification rate decreases. Although the computed results align with the analytical results from Abel's equation of the first kind and the conceptual analysis of transport processes involving three phases in different shapes, further confirmatory and quantitative experimental measurements are essential for validation.

## CRediT authorship contribution statement

**I.C. Hsieh:** Validation, Software, Methodology, Investigation. **P.T. Tseng:** Validation, Software, Methodology, Investigation. **W.T. Chen:** Validation, Software, Methodology, Investigation. **P.S. Wei:** Conceptualization, Methodology, Resources, Supervision, Validation, Writing – original draft, Writing – review & editing. **K.C. Liao:** Validation, Software, Methodology, Investigation.

## Ethics statement

All participants provided informed consent to participate in the study.

## Data availability statement

The data that support the findings of this study are publicly available within the article.

## Declaration of competing interest

The authors declare that they have no known competing financial interests or personal relationships that could have appeared to influence the work reported in this paper.

## Nomenclature

*C*: solute concentration, mol/ $m^{3}$
*D*: solute diffusivity, $m^{2}/s$
*f*: fraction, $f_{l}\left(T\right)=\left[1+\tanh\left(\left(T-T_{m}\right)/\Delta T_{m}\right)\right]/2$
*g*: gravity acceleration, $m/s^{2}$
*k*: thermal conductivity, W/m-K
*K*: Henry's law constant, Pa-$m^{3}$/mol
$k_{p}$: equilibrium partition coefficient
$L_{total}$: latent heat, J/kg
*p*: pressure, Pa
$R_{u}$: gas constant, J/mol-K
*t*: time, s
*T*: temperature, K
*u*, v: horizontal velocity component and velocity vector, m/s
$Vf_{g}$: gas fraction = (1−ϕ)*/2*
$Vf_{mat}$: condensed material fraction = (1+ϕ*)/2*
$V_{in}$: interface velocity, m/s
*x,y*: coordinates, m

**Greek letters**
ρ: density, $\text{kg}/m^{3}$μ dynamic viscosity, kg/m-s
ϕ: phase field function, ϕ = 1 and −1 for condensed material and gas
$\Delta T_{m}$: temperature range across melting temperature, K
κ: curvature = ∇⋅n, 1/m
χ: phase field parameter $\equiv \omega /\varepsilon^{2}$, m-s/kg
ω: mobility, $sm^{3}/\text{kg}$
σ: surface tension ≡$2\sqrt{2}\lambda /3\varepsilon$, N/m
γ: mole fraction of solute in surrounding gas
θ: contact angle, degree
ε: interface thickness, m
λ: mixing energy density, N

**Subscripts**
*g*, l: gas and liquid
*m*: melting point
*mat*: condensed material
*s*: solid
